# A Comprehensive Review of Magnetoencephalography (MEG) Studies for Brain Functionality in Healthy Aging and Alzheimer's Disease (AD)

**DOI:** 10.3389/fncom.2018.00060

**Published:** 2018-08-23

**Authors:** Pravat K. Mandal, Anwesha Banerjee, Manjari Tripathi, Ankita Sharma

**Affiliations:** ^1^Neuroimaging and Neurospectroscopy Lab, National Brain Research Centre, Gurgaon, India; ^2^Department of Neurodegeneration, Florey Institute of Neuroscience and Mental Health, Melbourne, VIC, Australia; ^3^Department of Neurology, All Indian Institute of Medical Sciences, New Delhi, India

**Keywords:** magnetoencephalography, mild cognitive impairment, Alzheimer's disease, functional connectivity, effective connectivity, network analysis, machine learning, multimodal imaging

## Abstract

Neural oscillations were established with their association with neurophysiological activities and the altered rhythmic patterns are believed to be linked directly to the progression of cognitive decline. Magnetoencephalography (MEG) is a non-invasive technique to record such neuronal activity due to excellent temporal and fair amount of spatial resolution. Single channel, connectivity as well as brain network analysis using MEG data in resting state and task-based experiments were analyzed from existing literature. Single channel analysis studies reported a less complex, more regular and predictable oscillations in Alzheimer's disease (AD) primarily in the left parietal, temporal and occipital regions. Investigations on both functional connectivity (FC) and effective (EC) connectivity analysis demonstrated a loss of connectivity in AD compared to healthy control (HC) subjects found in higher frequency bands. It has been reported from multiplex network of MEG study in AD in the affected regions of hippocampus, posterior default mode network (DMN) and occipital areas, however, conclusions cannot be drawn due to limited availability of clinical literature. Potential utilization of high spatial resolution in MEG likely to provide information related to in-depth brain functioning and underlying factors responsible for changes in neuronal waves in AD. This review is a comprehensive report to investigate diagnostic biomarkers for AD may be identified by from MEG data. It is also important to note that MEG data can also be utilized for the same pursuit in combination with other imaging modalities.

## Introduction

Magnetoencephalography is a non-invasive technique that measures oscillatory magnetic fields produced in the brain due to neuronal activity with excellent temporal and reasonable amount of spatial resolution (Cohen, 1968). At the cellular level, neurons have electrochemical properties that lead to the flow of electrically charged ions and subsequently generation of electromagnetic fields. The magnetic field generated by an individual neuron is very weak, but multiple neurons combined in a specific area produce a field that is measurable outside the head. This neuromagnetic field is in the range of 10–15 T (femtotesla, fT) for cortical activities. Magnetic field follows Ampere's “right-hand rule,” with the field directed outward on one side and inward on the other side of a tangentially oriented source current, forming a characteristic dipolar pattern in magnetic field sensors near the scalp. Radially oriented (with respect to the skull surface) source currents generate negligible magnetic field outside the head. Therefore, MEG is mainly sensitive to tangentially oriented sources in sulcal walls. Neuromagnetic fields are captured using highly sensitive superconducting sensors, called SQUIDs (Superconducting Quantum Interference Device). Activity from deeper cortical and subcortical areas is difficult to detect as they exist at longer distance from the sensors. SQUID systems are typically composed of two types sensors, called as magnetometer and gradiometer. Magnetometers measure magnetic field directly, and gradiometers as pairs of magnetometers positioned at a small distance form one another, calculate the difference in the magnetic field between their two locations, are used in MEG data acquisition (Cohen, 1972; Hämäläinen et al., 1993). These magnetic fields are produced simultaneously with electrical activity, MEG captures same millisecond resolution as EEG (Electroencephalography), allowing to examine neural activity at its natural temporal resolution. Thus, MEG provides a more direct measure of neuronal activity than functional magnetic resonance imaging (fMRI), which records blood-oxygen-level dependent (BOLD) responses.

Various MEG systems are available commercially such as (a) Elekta/Neuromag: 306 channel, (b) MAGNES: 148 channel, (c) CTF/VSM System: 275 channel (MISL), (d) Tristan Technologies, BabySQUID (for pre- and full-term infants), (e) MEGSCAN: 320 channel, (f) Mecurer, portable MEG system, (g) Yokogawa MEG: 160 channel (originally designed by Kanazawa Institute of Technology). Among all these systems, Eleckta Neuromag (Cichy et al., 2016b; Engels et al., 2016a; López et al., 2017a; López-Sanz et al., 2017), MAGNES Fernández et al., 2002, 2013; Maestú et al., 2003; Bajo et al., 2012; Poza et al., 2014; Gómez et al., 2017; Juan-Cruz et al., 2017, and CTF systems (de Haan et al., 2012c; Ranasinghe et al., 2014; Brookes et al., 2016a; Tewarie et al., 2016; Josef Golubic et al., 2017; Koelewijn et al., 2017) are mostly used. However, for clinical setting Elekta Neuromag and CTF/VSM systems are the popular ones.

Oscillatory brain signals are commonly categorized into five frequency bands: Delta (0.2–3 Hz), Theta (4–7 Hz), Alpha (8–13 Hz), Beta (14–31 Hz), and Gamma (32–100 Hz). Each band is associated with different physiological information involving brain activities. Delta (δ) waves are accompanied with deep levels of relaxation and restorative sleep. It has been found that δ waves are associated with unconscious tasks of the body. Irregular δ waves have been connected to awareness as well as learning difficulties (Gloor et al., 1977). Theta (θ) waves are mostly linked with sleep and it may occur during deep meditation also and it is the associated to memory, intuition, and learning (Klimesch, 1999). Alpha (α) waves are associated with resting state oscillations and awaken brain and it aids overall mental coordination, calmness, alertness, intelligence and cognitive abilities (Klimesch et al., 1998). Beta (β) waves are dominant during attentive cognitive task in normal conscious state. “Fast” activities of β waves are observed in concentration, decision making, anxiety and excitement (Teplan, 2002). Gamma (γ) waves are interrelated to simultaneous processing of information flow from various brain regions. γ frequency is above the neuronal firing range. Presence of γ is associated to cognizance (Tallon-Baudry and Bertrand, 1999). Among these existing band waves certain bands are associated with cognitive decline. MEG plays an important role for detecting early changes in these rhythms and provides prodromal features of AD. Early symptoms of AD includes progressive loss of cognitive functions. One of the major histopathological hallmarks of this disease is the formation of amyloid-beta plaques. Neurological changes in neurogenesis are evident already before plaque deposition and might contribute to well-known early dysfunctions in prodromal AD (Borroni et al., 2002; Ito et al., 2015; Unger et al., 2016).

Millions of people are affected worldwide by AD and this number is increasing every day (Wortmann, 2012). AD affects not only the individual but also it has serious impact the entire family of the patient as well as on society and economy. The actual cause of AD is not known yet, however, both clinical and laboratory research have revealed oxidative stress is related to AD. It is believed from available clinical data that hippocampal as well as frontal cortex regions, the glutathione level depletion is linked to the conversion from a healthy aged subject to MCI (Mandal et al., 2015). There are other associated features with hippocampal and frontal cortex texture, which also change in AD (Drachman, 2006). At present, MCI or AD are detected symptomatically by the clinicians and various neuropsychological tests like Clinical Dementia Rating (CDR) (Morris, 1993), Mini–Mental State Examination (MMSE) (Folstein et al., 1975). Seven minute screen (7MS) that consisted of four individual tests (orientation, memory, clock drawing, verbal fluency) (Solomon and Pendlebury, 1998), functional assessment staging (FAST) (Auer and Reisberg, 1997) and Montreal Cognitive Assessment (MoCA) test (Nasreddine et al., 2005) are used for screening of AD.

This review focuses on the MEG analysis techniques and modulation of neuronal rhythms in AD brain. Motivation of the review is to provide an outline for the application of MEG for connectivity analysis and its application combining other neuroimaging modalities that can help for the identification of early diagnostic biomarker for AD.

The manuscript is divided into seven sections. After introduction, in section The Schematic for MEG Signal Acquisition and Analysis, the comprehensive flow diagram of MEG data acquisition and analysis is presented. Section Resting State and Event-Related Response Studies describes about resting state and evoked studies followed by description of source reconstruction (section Source Reconstruction). The cutting-edge research of MEG analysis for AD involving single channel, connectivity and network analysis is scrutinized from existing literature in section MEG Data Analysis. Section Machine Learning Approach for MEG Based Analysis gives a brief idea about the application of machine learning algorithms on MEG data in AD population. Various methods of statistical analysis performed with MEG data is discussed in section Statistical Analysis of MEG Data. Section Discussion outlines the overall implementation and experimental outcomes of MEG studies. Section Conclusions summarized all the generally applicable procedures and points onto the scope for future research.

## The schematic for MEG signal acquisition and analysis

**Figure 1:**
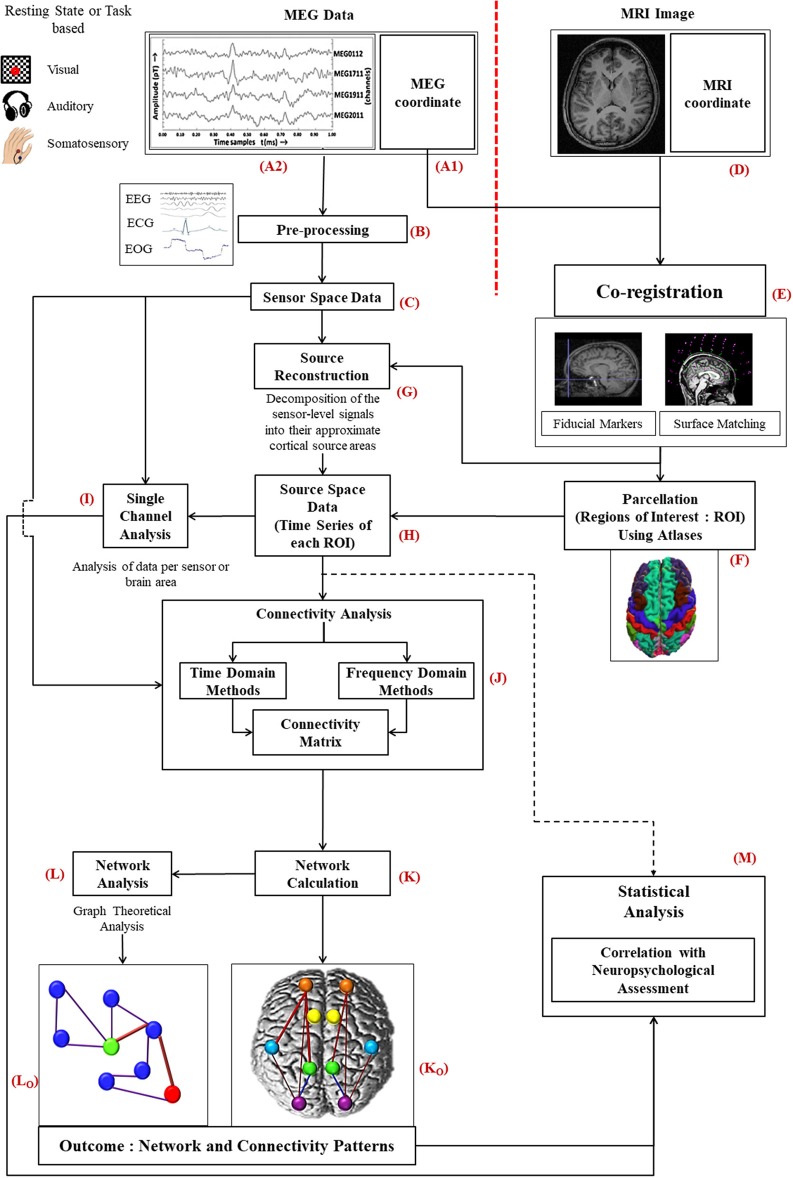
Diagrammatic representation of the work flow for MEG data analysis: MEG data acquired from subjects **(A2)** is preprocessed **(B)** to a filter noise from sensor space data **(C)**. Information of MEG co-ordinate **(A1)** is required with the MRI image **(D)** of the same subject for co-registration **(E)**. Source reconstruction of MEG data is performed thereafter **(G)**. Then the source space data **(H)**, time series generated for each ROI, is further processed for single channel **(I)**, and connectivity analysis **(J)**. To select ROIs parcellation is conducted using atlases **(F)**. After evaluating the network **(K)** using the connectivity information, graph theoretical network analysis is performed **(L)**. Statistical analysis is accomplished along with the information of neuropsychological assessment **(M)**.

The complex analysis procedure for MEG signals consists of various stages including co-registration with MRI images, forward and inverse problem as well as applicable steps for data analysis (Figure 1). Subjects participating in MEG study; also undergo MRI for 3D anatomical images.

The information related to MEG coordinate system (Figure 1A1) is registered by following two steps: (i) fiducial marking, where three anatomical landmarks, the nasion, the left preauricular point (LPA) and the right preauricular point (RPA), are indicated on the patient's head, and (ii) surface modeling, where head digitization is performed with a 3D tracker prior to the MEG examination (Hämäläinen et al., 1993). The fiducial points need to be determined accurately to minimize co-registration errors leading to point to point variation within the brain. Incorporation of new techniques can improve the precision and robustness of the co-registration process (Towle et al., 1993).

MEG co-ordinate system is computed before MEG signal acquisition with above explained digitization process. On the other hand, MRI co-ordinate system (Figure 1D) is generated after the MRI image acquisition. Raw MEG data (Figure 1A2) is acquired from the subjects in a magnetically shielded room either in resting condition (no task) or during certain tasks as per the study requirement. Processing scheme of MEG data are the same for resting state as well as with evoked potential conditions, however, only the execution scheme will vary. In resting state category, the subject needs to be fully awake as drowsiness may introduce noise. For event-related studies, the subject is asked to perform certain tasks in reaction to a given stimulus (e.g., visual, auditory, or somatosensory). Simultaneously with the recording of MEG signals, brain's electrical activity, heart's electrical activity and eye movements of the subject are captured by means of electroencephalogram (EEG), electrocardiogram (ECG), and electrooculogram (EOG), respectively.

The acquired MEG signal is visually observed and then processed for head movement correction and compensation is accomplished with built-in software for example Max filter in ELEKTA/Neuromag system, and commercially available other software like Brain Electrical source analysis (BESA), ASA, Curry, EMSE. The environmental interference and constant or periodic artifact correlation is performed by applying Single Space Separation (“SSS”) or temporal Single Source Separation (“t-SSS”). Single Space Separation is a new method for compensation of external interference and sensor artifacts by decomposing the MEG signal into inner source signal and outer noise. Independent Component Analysis (ICA) or template can also be used for rest of the biological artifacts like eye blinks. Pre-processing (Figure 1B) includes the following steps: Identifying nonfunctional (defective) channels followed by correction in head movement followed by Software interference suppression [Signal- space projection (SSP), Signal-space separation (SSS), T-Signal space separation (t-SSS)] and later artifact identification and rejection of environmental interferences and subject interference (cardiac activity, muscular activity; Velmurugan et al., 2014).

Moreover, at this stage of preprocessing of MEG data, filtering may also be applied to segment the MEG data into different band waves (δ, θ, α, β, and γ). Subsequently, the exclusive sensor space time series MEG data (Figure 1C) distributed over all the brain regions, which is processed further for single channel analysis (Figure 1I) and for connectivity estimation (Figure 1J) is generated.

Co-registration of MEG data with anatomical 3D MRI image (Figure 1E) enables source localization of the MEG data (Figure 1G). This source reconstructed MEG data, which is called source space data (Figure 1H), is used for connectivity estimation among the different selected regions of interests (ROI) as well as single channel analysis. Source reconstruction is performed for all the MEG sensors/broadband activity. Afterwards, source space time series is separated into different frequency bands.

Parcellation is implemented to divide the brain in different ROIs (Figure 1F). ROI selection is performed by parcellating the cortex using either source-sensor geometry approach or with native MRIs or utilizing various available atlases (Dickie et al., 2017). In MEG based studies automatic anatomical labeling (AAL) (Brookes et al., 2016; Engels et al., 2016a,b, 2017b; Hillebrand et al., 2016; Tewarie et al., 2016; Koelewijn et al., 2017; López et al., 2017a; Yu et al., 2017), Harvard Oxford (López et al., 2014a; López-Sanz et al., 2017) and Destrieux and Desikan-Killiany atlases have been implemented. Tools such as MarsBar in SPM, Featquery function in FSL, MRIcroN software, and ROInets are developed for ROI based analysis. The analysis for voxel time series within each ROI is achieved in various ways. In some studies, the voxel with maximum power within ROI represents the time series for corresponding ROI (López et al., 2017a). Researches also generate time series by calculating the weighted distance from center of mass of the respective region (Brookes et al., 2016). For specific representation of voxel, the centroid voxel was reported in some studies (Engels et al., 2017b; Yu et al., 2017). Maximum pseudoZ voxel is also used as alternative method to represent time series for specific ROI (Engels et al., 2016a).

Single channel data analysis (Figure 1I) includes various methods (e.g., spectral analysis, signal complexity etc.). For single channel analysis, sensor space data denotes data per sensor but in source space, it represents the analysis of data from a particular brain region. Keeping out anatomical links, brain connectivity can be divided into two classes: functional connectivity (FC) and effective connectivity (EC). For connectivity evaluation, MEG data is analyzed using time domain (nonlinear forecasting, cross mutual information etc.) as well as frequency domain parameters (coherence, phase locking value etc.) (Figure 1J). This analysis provides a correlation or adjacency matrix and the off-diagonal elements of that matrix represent different weights of connectivity between the corresponding ROIs. For source space MEG data, FC is the measure of co-modulation of separate sources (selected by ROIs) but for sensor space connectivity interpretation is difficult as it is not linked with a specific brain location. The data related to connectivity is utilized to build a linked brain network (Figure 1K). The different brain regions associated functionally or causally are visualized as connectivity patterns (Figure 1Ko). Various graph theoretical algorithms (Figure 1L) like clustering coefficient, path length etc. are applied for a global analysis of the brain network (Figure 1Lo).

Moreover, statistical analysis is performed for each method of analysis (single channel, FC, or network) along with the results from neuropsychological battery to evaluate the accuracy, sensitivity and specificity of the final outcome of the study (Figure 1M). The steps and related terminologies are explained later in this manuscript.

Various open source software and toolboxes are available for MEG data analysis. Brainstorm (Tadel et al., 2011), EEGlab (Delorme and Makeig, 2004), FieldTrip (Oostenveld et al., 2011), MNE (Gramfort et al., 2014), NutMEG (Dalal et al., 2004), OpenMEEG (Gramfort et al., 2010), SPM (Friston et al., 1994), EMEGS (electromagnetic encephalography software) (Peyk et al., 2011) etc. are commonly used. Commercial software packages are also available DANA and CURRY (NeuroScan, 2017). Though these tools keep updating on a regular basis but there remain some complications associated with MEG data processing and analysis.

## Resting state and event-related response studies

Resting state is defined as the stage when a subject is awake but not performing any task. MEG has been implemented for resting state network as well as evoked study in AD. Extra care must be taken to make sure that the AD patients are awake and alert as drowsiness affects activities of the resting state brain (Verdoorn et al., 2011).

It has been observed that the parts of brain responsible in the activation differs from resting state to task based conditions (Raichle et al., 2001). Brain regions that correlate in resting state are found to be involved in co-activation during tasks however their roles are likely to get shifted in evoked study (Daianu et al., 2013).

In evoked studies, association of any specific brain region with the task was correlated for probable AD and HC group (Berendse et al., 2000). The instant reduction of the signal amplitude after opening the eyes was found to be diminished in AD, but functional connectivity (FC) remained unaltered for eye opening or closing (Berendse et al., 2000). Multiple regions of brain activity are localized and also their time courses are characterized from somatosensory and visual activity task using multistart analysis of MEG data (Aine et al., 2000).

In memory based task, a relation between hippocampal atrophy and the degree of neurophysiological activity in the left temporal lobe has been demonstrated (Maestú et al., 2003). A reduced MEG response during the retention period of a working memory task was observed in AD and the findings were in line with MRS data (Maestu et al., 2005). Task based evoked potential analysis can also differentiate between AD and HC subjects but the clarification of changes may be difficult depending upon task performance and level of concentration of the subjects during experiment (Dickerson, 2007).

## Source reconstruction

MEG data is useful to get insight for brain functionality and the role of large-scale network is studied by approximation of neuronal interaction at source level. To accomplish this objective, reconstruction is performed for source time series. For source reconstruction, the forward and inverse modeling of MEG sensor data needs to be implemented with the help of MRI data. The forward problem is specified as the calculation of the magnetic field vector which is acquired outside the head. Inverse problem is involved in calculating the current density of the source that produce magnetic field vector. For source reconstruction of MEG data, the most commonly applied techniques found in the literature are: equivalent current dipole (ECD) (Kiebel et al., 2008), beamforming (Sekihara et al., 2001), and minimum norm estimation (MNE) (Hämäläinen and Ilmoniemi, 1994). Minimum current estimation (MCE) is also popularly applied for source estimation and localization of MEG data (Uutela et al., 1999).

Equivalent current dipole (ECD) has been used to analyze the magnetic counterparts of P50 and mismatch negativity for a MEG based study with passive oddball paradigm of AD patients. In comparison to HC subjects, larger cortical activation of standard-evoked M50 was observed in AD (Cheng et al., 2012). ECD has also been implemented for MEG based dipole density estimation of δ and θ band in AD (Fernández et al., 2003).

In studies utilizing beamforming procedure, it has been found that a frontal shift of α event-related synchronization (ERS) elicited by an eyes-open/eyes-closed paradigm indicating early changes in AD (Hincapié et al., 2017). This may represent a physiological state marker of the disease (Ishii et al., 2010). Application of beamformer based source reconstruction has been implemented upon resting state MEG signals from single-domain and multi-domain amnestic MCI (md-aMCI) subjects (Pineda-Pardo et al., 2014). The fiber densities between the regions, using diffusion-weighted MRI, defined the anatomical connectivity. Graphical Lasso (GL) has been used to estimate network architecture. Results of this study indicated that introduction of an anatomical prior knowledge might improve the expressivity of the model and, in most cases, leads to a better classification between groups (Pineda-Pardo et al., 2014).

Minimum norm estimation (MNE) has been used to detect synchronous and distributed neural activity of different cortical areas (Jensen and Vanni, 2002). MNE was applied for cortical origin estimation of δ band activity. Significant difference in δ band activity was reported for AD and HC groups from MEG data analysis (Fernández et al., 2013). MNE has also been applied for source reconstruction of MEG data in dementia and healthy individuals with subjective memory loss (Maestú et al., 2008).

Minimum current estimation (MCE) has been utilized to identify cortical sources of spontaneous brain oscillation from MEG data for MCI and AD. In comparison to HC group, oscillatory abnormalities in the alpha source distribution were clearly visible in AD whereas for MCI significant changes were not observed (Osipova et al., 2006).

## MEG data analysis

The MEG signal analysis is a complex procedure and is characterized in three categories: single channel analysis, connectivity analysis, network analysis (Engels et al., 2017a).

### Single channel data analysis

Single channel analysis is based on per channel local analysis and studies have been performed for the assessment of AD and control subjects from individual time series MEG data.

Single channel analysis studies have been executed to observe a distinction between healthy individuals and AD by time-frequency analysis of MEG data. Parameters reported in literature related to single channel analysis is classified into four groups: (i) spectral analysis, (ii) signal complexity, (iii) signal regularity, and (iv) signal predictability.

In comparison to healthy individuals, increase in absolute as well as relative power was perceived in slow frequency bands of δ and θ in AD, but decrease in these power values was observed in high frequency bands of α, β, and γ for AD. These results are observed in the parietal, temporo-parietal, posterior parietal areas as well as precuneus cortices and hippocampus (Fernández et al., 2002, 2003, 2006b, 2013).

Frequency analysis of MEG data has also been performed in various studies (Escudero et al., 2008, 2009). Peak frequency, mean frequency and median frequency were reported lower for AD compared to HC subjects (van Walsum et al., 2003; Poza et al., 2007b; Montez et al., 2009). It has been found that in AD, lower α band sources are predominant in the temporal regions, whereas in the controls, robust α sources were found near the parieto-occipital sulcus (Osipova et al., 2005). The activation within the parieto-occipital region was significantly weaker, and activation in the right temporal area was significantly enhanced in the AD (Osipova et al., 2005). Studies reported less complexity in AD by evaluating Lempel-Ziv complexity, fractal dimension and also correlation dimension (Gómez et al., 2009a,b; Hornero et al., 2009).

Nonlinear analysis has also been reported in MEG based studies (Abatzoglou et al., 2007; Escudero et al., 2009). Spectral entropy and ratio have also been examined from MEG data (Poza et al., 2007a, 2008b; Bruña et al., 2012). Approximate entropy, sample entropy as well as multiscale entropy values have been found to be lower in AD than HC subjects from MEG data analysis (Hornero et al., 2009). These results are consistent with the findings of MRI studies where reduction in entropy of hippocampus have been reported (Drachman, 2006).

Stationarity and equilibrium of signals from AD were found to be disrupted (Gómez et al., 2009b; Bruña et al., 2012) similar to complexity studies. Abnormal and predictable dynamics was reported in AD by observing low decrease rate of auto mutual information from MEG data (Gomez et al., 2006b; Gómez et al., 2007b; Hornero et al., 2009). Indication of more regular oscillations in AD than controls was found by high spectral crest factor and spectral turbulence and wavelet turbulence (Poza et al., 2008a, 2014). Comparatively high power in the lower frequency bands and low power in the higher frequency bands confirmed this information and relates to the studies involving EEG (Micanovic and Pal, 2014).

**Figure 2:**
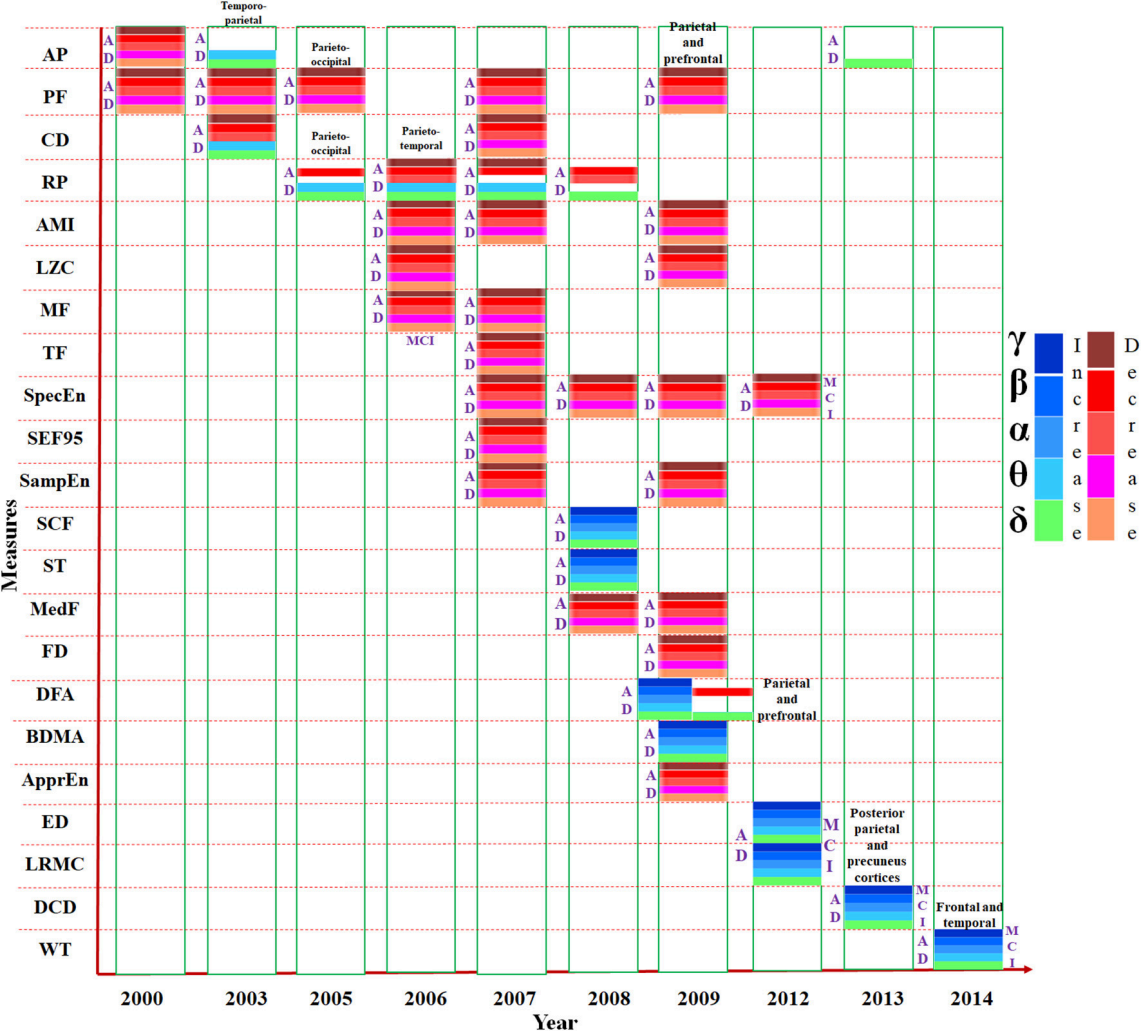
Presentation of various measures (e.g., mean frequency, relative power etc.) used in MEG single channel analysis, mentioned in the vertical axis, in chronological order. The horizontal axis represents the year of study. Changes to the parameters in the frequency bands (δ, θ α, β, and γ) are indicated using different color codes. AP, Absolute Power; PF, Peak Frequency; CD, Correlation Dimension; RP, Relative Power; AMI, Auto Mutual Information. LZC, Lempel-Ziv Complexity; MF, Mean Frequency; TF, Transition Frequency; SpecEn, Spectral Entropy; SEF95, 95% Spectral Edge Frequency; SampEn, Sample Entropy; SCF, Spectral Crest Factor; ST, Spectral Turbulence; MedF, Median Frequency; FD, Fractal Dimension; DFA, Detrended Fluctuation Analysis; BDMA, Backwards Detrended Moving Average; ApprEn, Approximate Entropy; ED, Euclidean Distance; LRMC, Lopez Ruis-Mancini-Calbet; DCD, Delta Current Density; WT, Wavelet Turbulence.

The changes observed in single channel analysis of MEG data using different parameters are represented in Figure 2. The increasing and decreasing measures of all the parameters, e.g., absolute power, approximate entropy etc., (listed in the vertical axis) for all the frequency bands (δ, θ, α, β, γ) have been shown in chronological order. Most of the studies have reported results common for all the frequencies in AD whereas some investigations informed significant difference in specific band waves. For example, relative power (RP) was reported increased explicitly in δ band but decreased in β band. Majority of the parameters implemented for single channel analysis of MEG data were reported reduced in AD compared to HC subjects though in few cases, metrics were found increased. The synthesis of single channel analysis of MEG studies, it is inferred that a slowing pattern of oscillations is visible in AD and MCI patients in frontal, parietal, temporal, and occipital brain regions.

### Connectivity analysis

The functionally connected areas of brain are explained with the help of connectivity analysis. This review discusses two types of connectivity analysis, FC and EC, for MEG data. FC is temporal correlation between remote neurophysiological events, and EC is defined as the influence one neural system exerts over another (Friston, 1994, 2011).

#### Functional connectivity assessment

Functional Connectivity (FC) is estimated in time as well as frequency domain using linear (e.g., correlation) and nonlinear (e.g., mutual information) methods (Sakkalis, 2011). MEG time series data of different brain areas that are functionally connected, are assumed to show a statistical relationship (Bastos and Schoffelen, 2016). MEG data analysis demonstrates the relationship between neural oscillations and functional connectivity of brain (Brookes et al., 2011). Path of information flow within the brain is understandable with the help of direction provided by FC (Blinowska, 2011).

Coherence is one of the widely used for FC estimation. Coherence is the degree of similarity of frequency components of two time series (of simultaneous values or leading and lagging relationships). Mathematically, coherence is the frequency domain equivalent to the time domain cross-correlation function. Its squared value quantifies, as a function of frequency, the amount of variance in one of the signals that can be explained by the other signal, or vice-versa, in analogy to the squared correlation coefficient in the time domain. The coherence coefficient is a normalized quantity bounded between 0 and 1.

(1) $$coh_{xy}()=\;\frac{\left|\frac{1}{n}\;\sum_{k=1}^{n}A_{x}\left(\omega ,k\right)A_{y\;}\left(\omega ,k\right)e^{i\left(\varphi_{x}\left(\omega ,k\right)-\varphi_{y}\left(\omega ,k\right)\right)}\right|}{\sqrt{\left(\frac{1}{n}\sum_{k=1}^{n}A_{x}^{2}\left(\omega ,k\right)\right)\left(\frac{1}{n}\sum_{k=1}^{n}A_{y}^{2}\left(\omega ,k\right)\right)}}$$

where, coherence of two signals *x* and *y* at frequency ω represented as coh_*xy*_ (ω) is computed using Equation (1), where *A*_*x*_, *A*_*y*_ are amplitude and φ_*x*_, φ_*y*_ are phase of signals *x* and *y*, respectively and *n* is the total number of data points. High value of coherence indicates strong functional connectivity.

Increase in coherence is reported in the δ band (Alonso et al., 2011; Escudero et al., 2011). On the other hand, loss of connectivity indicated by decreased coherence is found to be restricted in high frequency bands (Franciotti et al., 2006; Alonso et al., 2011). However, some studies reported no significance difference between AD and HC subjects by the evaluation coherence (Stam et al., 2002).

From coherence analysis, decreased neural connectivity of multiple brain regions including the right posterior perisylvian region and left middle frontal cortex correlated with a higher degree of disease severity has been reported (Ranasinghe et al., 2014). Insufficiency in executive control and episodic memory is correlated with reduced FC of the left frontal cortex, whereas visuospatial impairments is correlated with reduced FC of the left inferior parietal cortex (Ranasinghe et al., 2014). These results suggested that reductions in region-specific α-band resting state FC are strongly correlated with specific cognitive deficits in AD spectrum (Ranasinghe et al., 2014).

From the parameter named synchronization likelihood (SL), the strength of synchronization of two time series is evaluated based on state space embedding. Nonlinear forecasting (NF) and cross mutual information functions (CMIF) are measures for the predictability of one time series when a second series is known, have been implemented for FC analysis. Predictability based on similarity (based on the amplitude of two time series) has also been evaluated from MEG data using cross approximate entropy (Cr-appEn).

FC from MEG data was found to be lower in α, β, and γ bands but higher in θ band for AD (Stam et al., 2002, 2006) using SL. Increase in inter-hemispheric connections, decrease in anteroposterior FC was detected in MCI (Bajo et al., 2010). The inter-hemispheric increased synchronization values reflect a compensatory mechanism for the lack of efficiency of the memory networks (Bajo et al., 2010). Hence, these connectivity profiles support the idea of calling MCI as a “disconnection syndrome” partially (Bajo et al., 2010).

MEG study with a memory task has been performed on MCI group to characterize patients who would eventually go on to develop the disease (Bajo et al., 2012). It has been reported that progressive patients showed a differential profile of FC values compared with those patients who remained stable over time (Bajo et al., 2012). Time series was found more predictable for AD by the nonlinear forecasting approach (Gómez et al., 2008). While cross mutual information values were reported increased in AD (Alonso et al., 2011), cross-approximate entropy was observed decreased indicating synchronization better than HC subjects (Gómez et al., 2012). For the analysis of FC, variance information in source space projected Hilbert envelope time series has been extracted that has given important spatial information about functional relevance (Hall et al., 2013).

Another measure of connectivity is phase locking value (PLV). It is calculated using the instantaneous phase difference between a pair of signals. PLV for each data segment is calculated as the norm of the average vector for the pair of signals k and l from Equation (2)

(2) $$PLV_{k,l}=\;\left|\frac{1}{T}\sum_{t}e^{-j\left(\varphi_{k}\left(t\right)-\varphi_{l}(t)\right)}\right|$$

where, φ_*k*_(*t*) is the instantaneous phase of signal *k* at instant *t*, and *T* is the number of temporal points per segment.

Another metric used for FC estimation from MEG data is phase lag index (PLI), which evaluates the distribution of phase differences across observations. In terms of PLI, FC was observed lower in AD in α and β band (Stam et al., 2009). FC alterations based on PLV were found in both subjective cognitive decline (SCD) and MCI groups compared to HC subjects (López-Sanz et al., 2016, 2017). A hyper synchronized anterior and posterior network, characterized by a decrease in FC, was spotted in AD (López-Sanz et al., 2016, 2017). This decrease was more pronounced in the MCI group. These results indicate that SCD can be considered as a preclinical stage of AD (López-Sanz et al., 2016, 2017). FC was assessed using the amplitude envelope correlation was significantly lower in all-to-β cross-frequency coupling (CFC) in AD in the left hippocampus and several regions of the DMN (Engels et al., 2016b). Virtual electrodes have been used to correlate functional interactions with the slowing activity of hippocampi and cortical areas (Engels et al., 2016a). A virtual electrode is an estimate of the time-series of neuronal activity at a particular location in the brain. Estimating neuronal activity at the source level requires solving the inverse problem, i.e., the estimation of activity on the basis of the extracranial sensor recordings (Hillebrand and Barnes, 2005). In this study beamforming technique is used to solve this inverse problem. A decreased connectivity in AD, specifically in parieto-temporal areas in the α and β band in whole brain during resting state (Koelewijn et al., 2017).

The interfering factors to be considered in MEG data analysis are field spread (FS) and volume conduction (VC). Because of the topographical representation of magnetic field beyond the source, signal can be picked up at some distance and it is termed as field spread (Silva Pereira et al., 2017). Due to FS, a signal from one underlying source can be present in multiple time series. This creates error in the estimation of statistical dependencies between time series data which in turn hamper the evaluation of FC (Bastos and Schoffelen, 2016).

Posterior-to-anterior information flow over the cortex in higher frequency bands in HC subjects with a reversed pattern in the θ band has been reported (Engels et al., 2017b). The information flow from the precuneus and the visual cortex, toward frontal and subcortical structures, was found to be decreased prominently in AD (Engels et al., 2017b). MEG based multiplex brain network yields to an effective structure for the integration of the frequency specific networks (Yu et al., 2017).

#### Effective connectivity assessment

Recent studies have been performed on MEG data for the analysis of EC in AD and MCI (Gómez et al., 2017). Granger Causality (GC) was implemented for the estimation of EC. According to GC, if a signal P “Granger-causes” (or “G-causes”) a signal Q, then past values of P should contain information that helps to predict Q, above and beyond the information contained in past values of Q alone (Granger, 1969). For all the five conventional frequency bands, connectivity values were lower for MCI in comparison to HC subjects. Interhemispheric GC was found to be decreased between frontal areas (both directions) in θ, α, and β, from left central to right central in θ, between posterior brain regions in α and β, and finally between temporal areas (from right to left in θ, and in both directions in α, β, and γ; Gómez et al., 2017). On the other hand, statistically significant differences in intra-hemispheric couplings were found mainly between frontal, lateral, and posterior areas, but also from these aforementioned areas to central regions, and between posterior and frontal regions (Gómez et al., 2017). In θ, β, and γ bands, decrements in EC patterns was observed, whereas increments in EC was found in the δ band in AD using GC (Juan-Cruz et al., 2017).

After analyzing all the findings reported for MEG based connectivity studies, a decrease in MEG based functional connectivity in the higher frequency bands are observed. Increase in connectivity has mainly been observed in the parietal and temporal regions as well as between the parietal and occipital regions and here FC was not frequency dependent. It is concluded from all these connectivity analyses that parietal areas along with left frontal and occipital areas are associated with the decrease in long term connection and for short term connection involvement of the frontal and parietal regions of right hemisphere was reported.

**Figure 3:**
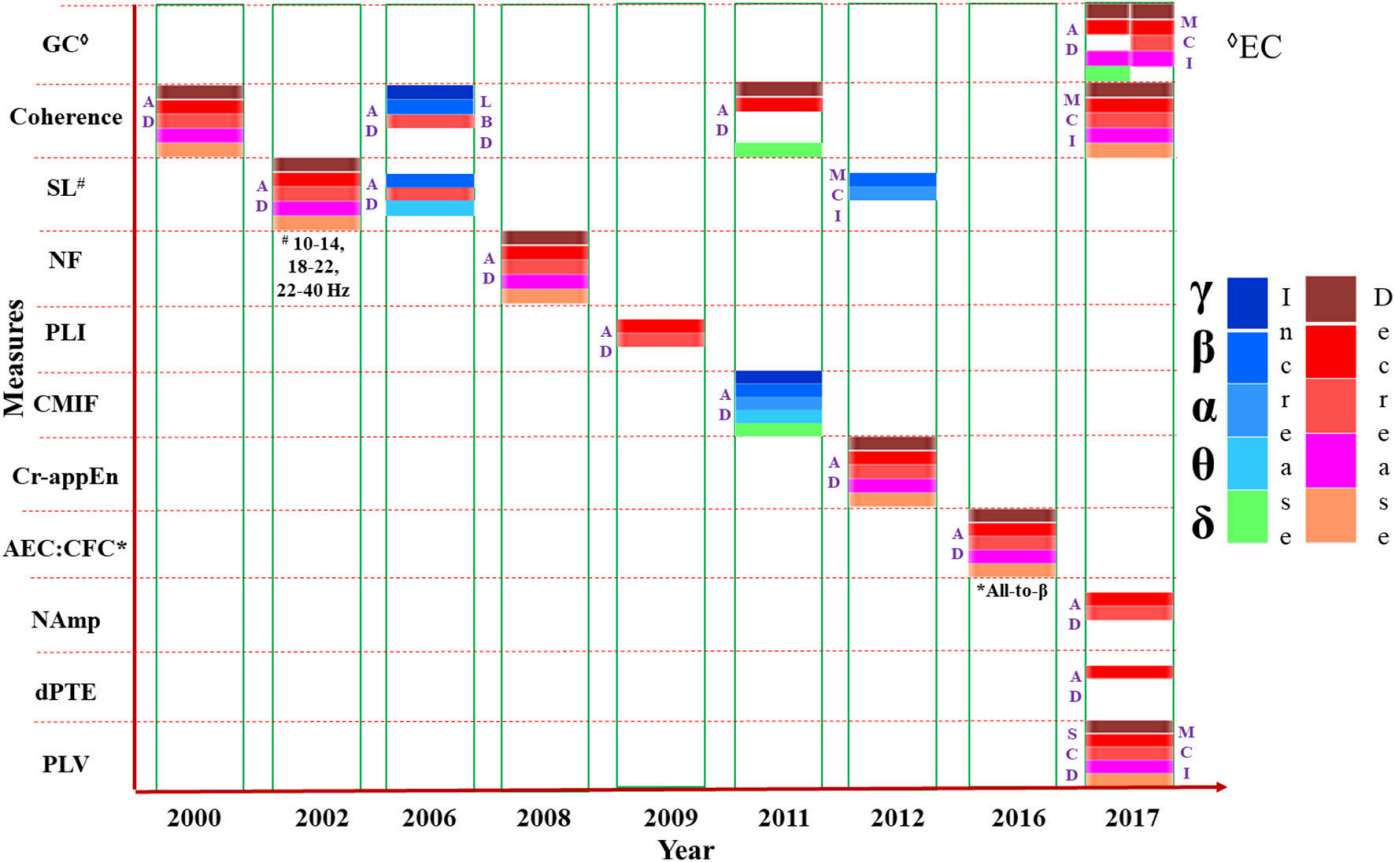
Presentation of various measures (e.g., coherence, SL etc.) used in connectivity analysis from MEG data, mentioned in the vertical axis, in chronological order. The horizontal axis represents the year of study. Changes to the parameters in the frequency bands (δ, θ α, β, and γ) are indicated using different color codes. GC: Granger Causality, Coherence; SL, Synchronization Likelihood; NF, Nonlinear Forecasting; PLI, Phase Lag Index; CMIF, Cross Mutual Information Function; Cr-appEn, Cross-Approximate Entropy; AEC, Amplitude Envelope Correlation; CFC, Cross Frequency Coupling; NAmp, Node based Amplitude; dPTE, Directed Phase Transfer Entropy; PLV, Phase Locking Value.

The pattern changes of connectivity in AD and MCI have been shown year wise from literature as compared to HC subjects in Figure 3. All the parameters (e.g., coherence, SL etc.) are listed in the vertical axis. Most of the studies have accomplished FC analysis of MEG data for AD and reported a decreased connectivity in all bands (δ, θ, α, β, and γ). Mostly the variations were observed in α and β bands. Although majority of the investigations inferred reduced connectivity than HC subjects, but variability of research outcomes are observed in different research groups. EC experiment was implemented only in two studies reported in 2017 which also detected less connectivity for AD and MCI.

### Network analysis

Brain networks deal with the modular and hierarchical nature of brain activities with the hub regions of brain (Bassett and Bullmore, 2009; Bullmore and Sporns, 2009). Graph and modern network theory applied on FC values has been used to find out the framework of brain topology. From connectivity matrix, graph theoretical algorithm based brain networks are derived. In order to capture most significant connections within a graph, the application of statistical thresholding scheme and selection of an appropriate filtering for meaningful topologies is a critical step in brain connectivity analysis. Common measures like clustering coefficient, Eigen vector centrality, characteristic path length, intramodular connectivity have been utilized for MEG based network analysis in AD research (Stam et al., 2009; Engels et al., 2017a). MEG based networks contain some organizational properties that is termed as “small-world topology” (Stam, 2004) to include high global efficiency as well as local interconnectedness (Watts and Strogatz, 1998). “Hub” is defined as a network node (brain region) with the specific property of having a high centrality, i.e., it plays an important role within the network. Many studies have been reported for the identification of the hubs in the brain (de Haan et al., 2012c). Highly localized clustering and short characteristics path length has been found in undirected binary and weighted brain networks (Rutter et al., 2013; Bassett and Bullmore, 2017).

MEG research in AD indicated decrease in hub regions in AD patients, specifically in the parietal region in the θ band (de Haan et al., 2012c). Lower FC is a result of weakening of hubs as these hubs are considered as the connectors of groups of highly functional intra-connected brain areas, or modules (De Pasquale et al., 2016). Inter-modular connectivity was found lower in AD compared to controls using MEG study (de Haan et al., 2012c). A relationship between network characteristics and performance on cognitive tests was observed in studies (Ortiz-Alonso et al., 2003). Lower hubness, which was measured with the Eigen vector centrality, has been correlated positively with lower MMSE, and lower between-module strength was related to poorer performance for word recall, word fluency and visual recognition (de Haan et al., 2012b,c). These results validate the concept of disintegration of functional connections and network disorganization in AD.

To avoid any spurious connection in a fully connected graph requires statistical filtering and surrogate networks are applied to address this problem. Surrogate network for normalization as well as comparison with random network has also been used in few studies (Stam et al., 2009; van Wijk et al., 2010; Rutter et al., 2013). Topological filtering (Dimitriadis et al., 2017a,b) is the next step to be followed to derive meaningful network structure consisting of only the essential interactions. Some approaches involving topological filter have been undertaken in network analysis for AD research, like minimum spanning tree (MST) (Çiftçi, 2011; López et al., 2017a), which is better than conventional procedure (Hillebrand et al., 2012). MST is a subset of the edges of a connected, edge-weighted (un) directed graph that connects all the vertices together, without any cycles and with the minimum possible total edge weight (Tewarie et al., 2015). MST is gaining popularity as it is assumption free and unbiased method. This method overcomes the constraints of existing topological filtering techniques by preserving the connectedness of brain network but also it typically ends up with large sparse graph. To address this problem, orthogonal MST (OMST) was introduced by employing different algorithms such as Kruskal (1956) and Prim (1957) to construct the MST for a weighted graph. The OMST method conserves the main advantage of MST and further confirms a denser and potentially more meaningful network. Other than OMST, the additional topological filtering techniques are global cost efficiency (GCE), mean degree, proportional, absolute and data driven based algorithms (Dimitriadis et al., 2017a,b). In MEG based resting state connectivity study for multi group GCE has been implemented (Dimitriadis et al., 2017a). A k-core network which is evaluated by thresholding the network to retain only those nodes with high nodal degree has been implemented for the construction of fully connected graph (Daianu et al., 2013).

In AD, neural complexity was found lower in low frequencies (van Walsum et al., 2003). Low clustering coefficient and short characteristic path length suggested a more random configuration of network in AD than HC subjects (Stam et al., 2009). Decrease in Eigen vector centrality reported the loss of a known temporal hub in AD and also fewer modules with weaker connections (de Haan et al., 2012b,c).

In MCI, an enhancement of the strength of connections, together with an increase in the outreach parameter was observed in the graphs using complex network analysis of MEG data with SL (Buldú et al., 2011). It indicates that memory processing in MCI is associated with higher energy expenditure and a tendency toward random structure, which breaks the balance between integration and segregation (Buldú et al., 2011). These reports suggest that for healthy individual's parietal region acts as a hub region.

**Figure 4:**
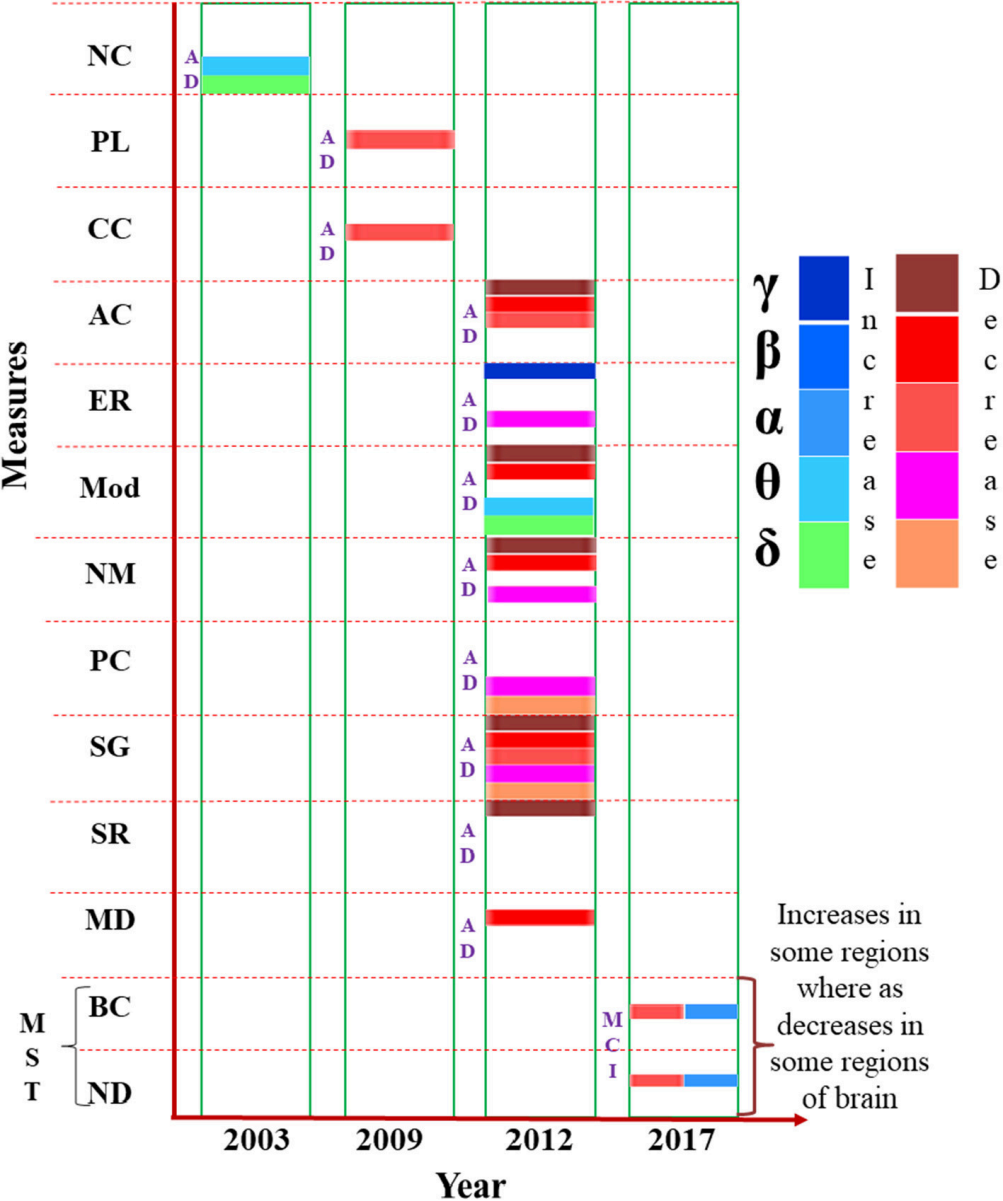
Presentation of various measures (e.g., node modularity, path length etc.) used in brain network analysis from MEG data, mentioned in the vertical axis, in chronological order. The horizontal axis represents the year of study. Changes to the parameters in the frequency bands (δ, θ α, β, and γ) are indicated using different color codes. NC, Neural Complexity; PL, Path Length; CC, Clustering Coefficient; AC, Algebraic Connectivity; ER, Eigen Ratio; Mod, Modularity; NM, Number of Modules; PC, Participation Coefficient; SG, Spectral Gap; SR, Spectral Radius; MD, Within Module Degree; MST, Minimum Spanning Tree; BC, Betweenness Centrality; ND, Node Degree.

Findings of MEG based FC analysis has been reported as small-world network within human brain characterized by dense local clustering for neighbor nodes along with a short path length between any pair of nodes (Stam, 2004; Stam et al., 2009). Source-space weighted functional networks were characterized with graph theoretical measures in event-related network analysis (ERNA) for a MEG based cognitive study. Dense and clustered connectivity between the hubs belonging to different modules is reported as the “network fingerprint” of cognition (Bola and Sabel, 2015). Recently, a new approach for MEG analysis is introduced and named as “multiplex network analysis” (Yu et al., 2017). Diagrammatic representation of the results reported for global brain network analysis performed using MEG data in a successive year-wise manner in Figure 4.

## Machine learning approach for MEG based analysis

The developments and availability of various feature extraction and classification techniques based on machine learning have been growing for MEG signal analysis (Deo, 2015). In combination with network analysis studies, implementation of machine learning algorithms like random forest, support vector machine (SVM) could easily distinguish the two clinical groups (Cichy et al., 2016b; Zanin et al., 2016). In a group study consisting subjects of multiple sclerosis, AD, schizophrenia, Sjogren's syndrome, chronic alcoholism, facial pain as well as HCs (Georgopoulos et al., 2007), MEG data was classified using Genetic algorithm-linear discriminant analysis (GA-LDA) with autoregressive integrated moving average (ARIMA) features. They reported results by dividing subjects into three sample groups, (1) 52 subjects, 6 groups, (2) 46 subjects, 5 groups, and (3) 142 subjects, 7 groups. Another study reported to classify AD and control group 70.73 and 78.05% accurately using features of SampEn and LZC, respectively with leave-one-out-cross validation method (Gómez et al., 2009a). However, they were able to achieve 85.37% accuracy by implementing adaptive network based fuzzy inference system classifier for distinguishing AD and control subjects. In a combined MEG and fMRI study, multivariate analysis of MEG data with linear SVM was used to select MEG sensors that contain discriminative information in noisy data without human intervention (Cichy et al., 2016a).

In a recent study (Maestú et al., 2015), a new approach of classification called Clinical Data Partitioning (CliDaPa) algorithm has been incorporated to distinguish MCI from controls. They proposed CliDaPa process that includes Chi Square filtering feature selection and machine learning classifiers. Random Forest (Breiman, 2001), Bayesian Network (Buntine, 1991), C4.5 induction tree (Quinlan, 1993), K-nearest Neighbor (Cover and Hart, 1967), Logistic Regression (Ng and Jordan, 2002), and SVM (Suykens and Vandewalle, 1999) classification algorithms are included in their proposed method. They applied bootstrap validation to measure the robustness and accuracy of proposed pipeline. High accuracy up to 86% was achieved by using 10-fold cross validation based Linear discriminant analysis (LDA) and radial basis function SVM (rbf-SVM) classifier for single domain and multi domain MCI patients. LDA was utilized for the classification of with leave-one-out cross validation (Bruña et al., 2012). Discriminant analysis has also been used for AD and HC classification (Fernández et al., 2003).

## Statistical analysis of MEG data

Parametric, e.g., students *t-*test, analysis of variance (ANOVA), Chi-Squared tests (Franciotti et al., 2006; Stam et al., 2006; Gómez et al., 2007b; Poza et al., 2007b, 2008a; Ishii et al., 2010; Engels et al., 2017b; López et al., 2017a; López-Sanz et al., 2017) as well as non-parametric, e.g., Wilcoxon–Mann–Whitney tests (Alonso et al., 2011) statistics has been applied for MEG data analysis (Kiebel et al., 2005). Parametric tests are performed depending upon specific assumptions.

Analysis of variance (ANOVA) and two-tailed *t-*test were applied to test the group differences. Spearman's bivariate correlation test were used to access the connection between cognitive status and network derived measures (Stam et al., 2009). Use of Systat software for windows has been reported to calculate Huynh-Feldt-corrected P values for MEG data (Stam et al., 2002). They stated application of a two-way repeated measure analysis of variance with AD and HC group as an inter subject factor and 117 MEG channels as intra subject factor. Pearson correlation coefficient has been used to correlate MEG and MRI volumetric variables (relative left and right hippocampal volume) to distinguish AD and HC group (Maestú et al., 2003). The AD vs. HC analysis has also been done using Multivariate analysis of variance (MANOVA) where they choose parameters correlation dimension and neural complexity (van Walsum et al., 2003). A three-way repeated measures ANOVA was used to compare power values between groups. To compare activation within ROIs and peak frequencies also *t-*test was carried out (Osipova et al., 2005). ANOVA with Greenhouse-Geisser correction has been utilized for the qualitative statistical classification among AD, MCI, and control groups (Escudero et al., 2011).

Non-parametric test, on the other hand, does not make any such assumption and can be performed even without any information regarding population or have a small population. Non-parametric two-tailed Mann–Whitney *U*-test was carried out using SPSS software for evaluation of statistical significance of classification between AD and normal individuals (Poza et al., 2007a). Mann–Whitney *U*-test has also been used with leave-out cross-validation to measure the ability of median frequency and spectral entropy to differentiate AD from HCs (Escudero et al., 2008). Kruskal–Wallis test was performed for each channel pair between MCI and HC (Bajo et al., 2010). For evaluation of statistical significance Wilcoxon signed rank test was performed on simulated data (Brookes et al., 2011).

The advantage of non-parametric statistical test is that it gives complete freedom of choosing the experimental conditions for comparison. This independence delivers an up-front approach to explain the Multiple Comparisons Problem (MCP) (Maris and Oostenveld, 2007). MCP is a commonly found problem in statistical analysis of MEG. This problem initiates since MEG-data are multidimensional. The signal is sampled at multiple channels and multiple time points; hence MEG-data becomes multidimensional. Moreover, during MEG data analysis the effect of interest (i.e., a difference between experimental conditions) is evaluated at an extremely large number of (channel, time)-pairs which in turn gives rise to MCP.

For correction of MCP, Bonferroni correlation was used while statistically validating MEG-MRS combined study using ANOVA for AD and HC (Maestu et al., 2005). In a comparative study of progressive MCI, stable MCI and controls using synchronization likelihood parameter Kruskal–Wallis (KW) test was performed. To correct for MCP, they applied non-parametric permutation testing followed by surrogate t-maps (Bajo et al., 2012). One study has done correction for MCP by taking maximum mean PLI values over ROIs (Hillebrand et al., 2012). Distribution for *t-*test was derived from permutation to avoid family wise error occurring due to MCP in the classification of healthy aged persons from MCI and AD (Fernández et al., 2013) and similarly to distinguish among sd-aMCI, md-aMCI, and HCs (López et al., 2014b). Implementation of false discovery rate (FDR) for multiple comparisons correction was not found significant in a *t*-test based classification of single domain amnestic MCI and HC group (Pineda-Pardo et al., 2014) whereas in other studies FDR showed robustness in correcting MCP (Ranasinghe et al., 2014; Engels et al., 2016b; Gómez et al., 2017). Mann–Whitney *U*-tests modified for multiple comparisons by a Bonferroni correction were performed in the statistical analysis of MEG based study of HC, MCI, and AD (Poza et al., 2014). Significant differences in FC between progressive MCI, stable MCI, and HCs was calculated using Mann–Whitney *U*-test to correct MCP using non-parametric permutation test (López et al., 2014a). Maximum *t-*value across ROIs of each permutation was utilized to build a distribution of maximum *t*-values to address MCP problem in a study of AD and HCs using MEG (Engels et al., 2016a). Permutation tests for random-effects inference and MCP correction has been performed with a cluster-level in MEG data analysis (Cichy et al., 2016b). Tukey's Honestly Significant Difference (HSD) MCP correction was implemented for neuropsychological scores in MEG based classification of aged HCs, SCD and MCI group (López-Sanz et al., 2016).

Although, in some studies both parametric as well as non-parametric statistics have implemented for comparison, but they have not applied MCP correction (de Haan et al., 2012c; Josef Golubic et al., 2017; Juan-Cruz et al., 2017).

## Discussion

MEG provide means to uncover AD related deviations in brain oscillations. In-depth analysis of MEG application in AD can be useful to identify plausible biomarkers to detect the early stages of this disease. This section entails thorough explanation about the outcomes of single channel, connectivity, network analysis of MEG data in AD and MCI.

The neuropsychological studies involving MMSE and FAST results of AD patients have been related to slowing of brain oscillations and cognitive declined indicated by MEG (Fernández et al., 2006a, 2013). Studies also found that low MMSE score in AD was related to slowing in parietal and central regions of brain (de Haan et al., 2008).

From the analysis of all the research reporting single channel analysis, it is evident that AD patients have less complex, more regular and predictable brain oscillations. It is the indication of the slowing oscillatory activities (Stam, 2005). Word processing in AD has also been examined using MEG data (Walla et al., 2005). Moreover, studies performing evaluation of wavelet turbulence and entropy analysis demonstrated an increase in the average degree of similarity within time series with the progress of AD (Poza et al., 2007b). Basically it indicates that in AD brain signals have a less uniform spectral control (Poza et al., 2007b). Nonlinear findings suggested that the decrease in complexity of brain signals in AD might be simply due to the altered spectrum reported in AD (Stam, 2005). Studies also pointed out that the complexity, regularity and predictability measures of the single channel MEG analysis are not entirely dependent on the spectral component of the data. Presence of nonlinear structures has been found from surrogate data of EEG and MEG in AD and HC subjects (Pereda et al., 2005; Gómez et al., 2009a). The effect of randomization was different in AD compared to controls which is an indication that complexity and regularity measures in AD studies cannot be completely attributed to the slowing of the signal. This aspect was also found in non-overlapping spectral and complexity study of MEG data (van Walsum et al., 2003). The actual reason for the decrease in complexity is not clear yet. However, loss of neurons and synapses and reduction in neurotransmitters might be involved in this process (Gómez et al., 2009a). It has also been reported that FC and brain network changes due to the changes in synaptic levels (de Haan et al., 2012b).

To properly describe the fact that most active regions have been observed with the most abundant changes in AD, an activity dependent degeneration hypothesis has been proposed (de Haan et al., 2012a). In a recent MEG study, changes in the activation of prefrontal brain area has been observed in early stage AD (Song et al., 2014).

Minimum-variance pseudo-unbiased reduced-rank estimation (MV-PURE) framework has been proposed for better reconstruction of source activity from MEG data (Piotrowski et al., 2017). In addition, multilayer network analysis approach has also been applied to integrate multiple frequency bands in a single framework and results reported disruption of hub regions in AD (Brookes et al., 2016).

MEG studies suggest that the parietal and temporal regions of the brain play an important role in brain functioning. A MEG based study of progressive MCI patients predicted that the increase in phase synchronization between the right anterior cingulate and temporo-occipital areas, is useful to understand the conversion from MCI to AD (López et al., 2014a).

Reports suggest that in MCI and AD difference in these regions are observed as compared to healthy subjects. In case of frequency as well as FC analysis, these regions have shown slow brain activity and decreased connectivity, respectively.

**Figure 5:**
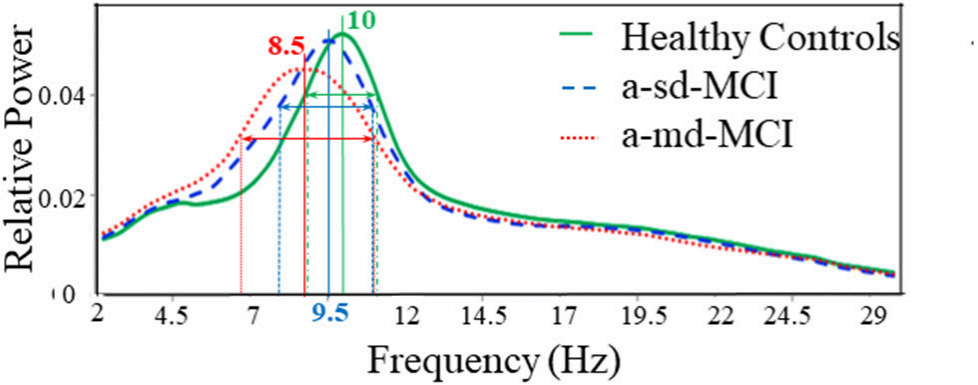
Average relative power spectra for all MEG channels in HC subjects (green line), the amnestic single domain (a-sd) MCI patients (blue dashed line) and the amnestic multi domain (a-md) MCI patients (red dotted line). This Figure is taken from literature (López et al., 2014b) with due permission of the American Aging Association and modified.

In a study administered by López et al. (2014b), oscillatory brain activities of subtypes of amnestic MCI have been compared by the relative power values of their MEG data. Total 105 subjects' data has been recorded in eyes closed resting state condition. Among all the subjects 36 were HC subjects, 33 were single domain amnestic mild cognitive impairment (sd-aMCI) and the rest 36 were multi-domain amnestic mild cognitive impairment (md-aMCI) patients. The average relative power in the frequency range of 1–30 Hz for each group has been calculated. Increase in relative power in lower frequency bands and decrease in power values in higher frequency bands has been observed in both MCI groups in comparison to control group. Prominent difference has been seen between two subtypes of MCI, the md-aMCI group showed a significant power increase within δ and θ ranges and reduced relative power within α and β ranges. In HC subjects a maximum value has been observed at 10 Hz whereas frequency peak for sd-aMCI and md-aMCI patients were found at 9.5 and 8.5 Hz, respectively (as shown in Figure 5). In addition to that different spectral distribution was visible among the groups. Indicating lower variability, a comparatively narrower band was found for HC subjects. A broader spectrum has been found for both the MCI groups. It might be a sign of higher variability and tendency to lower frequency peaks across subjects. These relative power values were correlated with neuropsychological tests scores and hippocampal volumes.

Changes in FC have been found in elders with SCD as well as MCI patients compared to healthy individuals in an investigation (López-Sanz et al., 2017). MEG data has been collected in resting state from 39 healthy control elders, 41 elders with SCD, and 51 MCI patients. FC has been evaluated based on source reconstructed MEG data using PLV.

**Figure 6:**
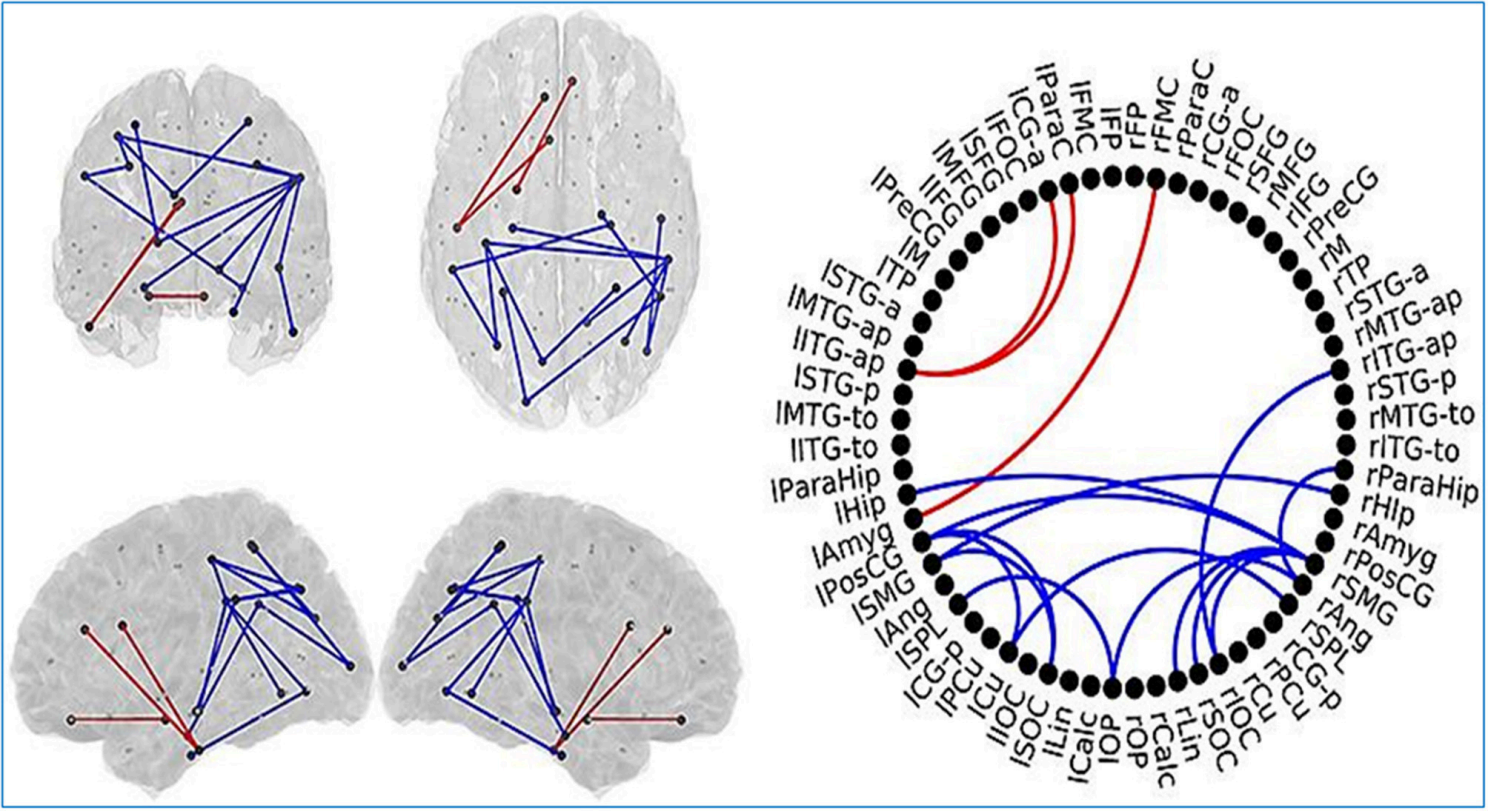
Links (connection among various brain regions) with significantly different FC values have been shown for comparative analysis. Left: Posterior, superior, left and right views of the brain. Right Circle plot shows a schematic view of the significant links. Comparison between healthy control (HC) subjects and mild cognitive impairment (MCI) group. Red lines indicate an increased FC value in MCI with respect to HC subjects. Blue lines indicate a decreased FC value in MCI with respect to HC subjects. This Figure is taken from literature (López-Sanz et al., 2017) with permission.

The anterior network presented higher FC in the MCI group compared to HC subjects in three links connecting anterior regions, including left inferior temporal gyrus, left paracingulate, and left anterior cingulate. The posterior network exhibited lower FC in the MCI group, and comprised 14 links between connecting posterior cortical structures such as: temporal medial structures (both hippocampi and right parahippocampus), parietal (left postcentral gyrus, both supramarginal gyri), and occipital areas (left frontal pole, both superior occipital cortices, right inferior occipital cortex, right lingual cortex) (Figure 6).

**Figure 7:**
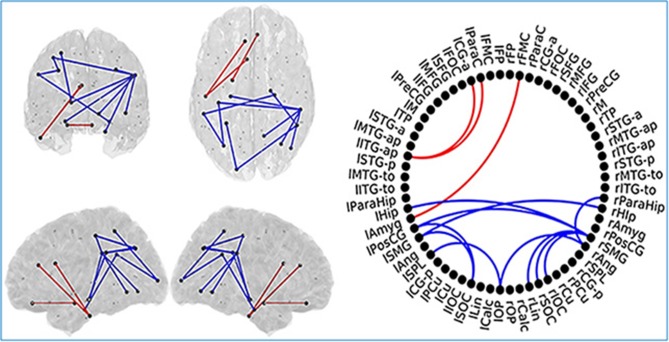
Links (connection among various brain regions) with significantly different FC values have been shown for comparative analysis. Left: Posterior, superior, left and right views of the brain. Right Circle plot shows a schematic view of the significant links. Comparison between healthy control (HC) subjects and subjective cognitive decline (SCD) group. Red lines indicate an increased FC value in SCD respect to HC subjects. Blue lines indicate a decreased FC value in SCD respect to HC subjects. This Figure is taken from literature (López-Sanz et al., 2017) with permission.

SCD subjects showed increased FC values respect to HC subjects in the same regions described in the previous comparison. SCD subjects also showed decreased FC in 11 links. Those links connected both intra and inter-hemispherical areas between posterior regions (as shown in Figure 7). Interestingly, all the links affected in the SCD group, were also disrupted in the MCI group in a similar manner.

**Figure 8:**
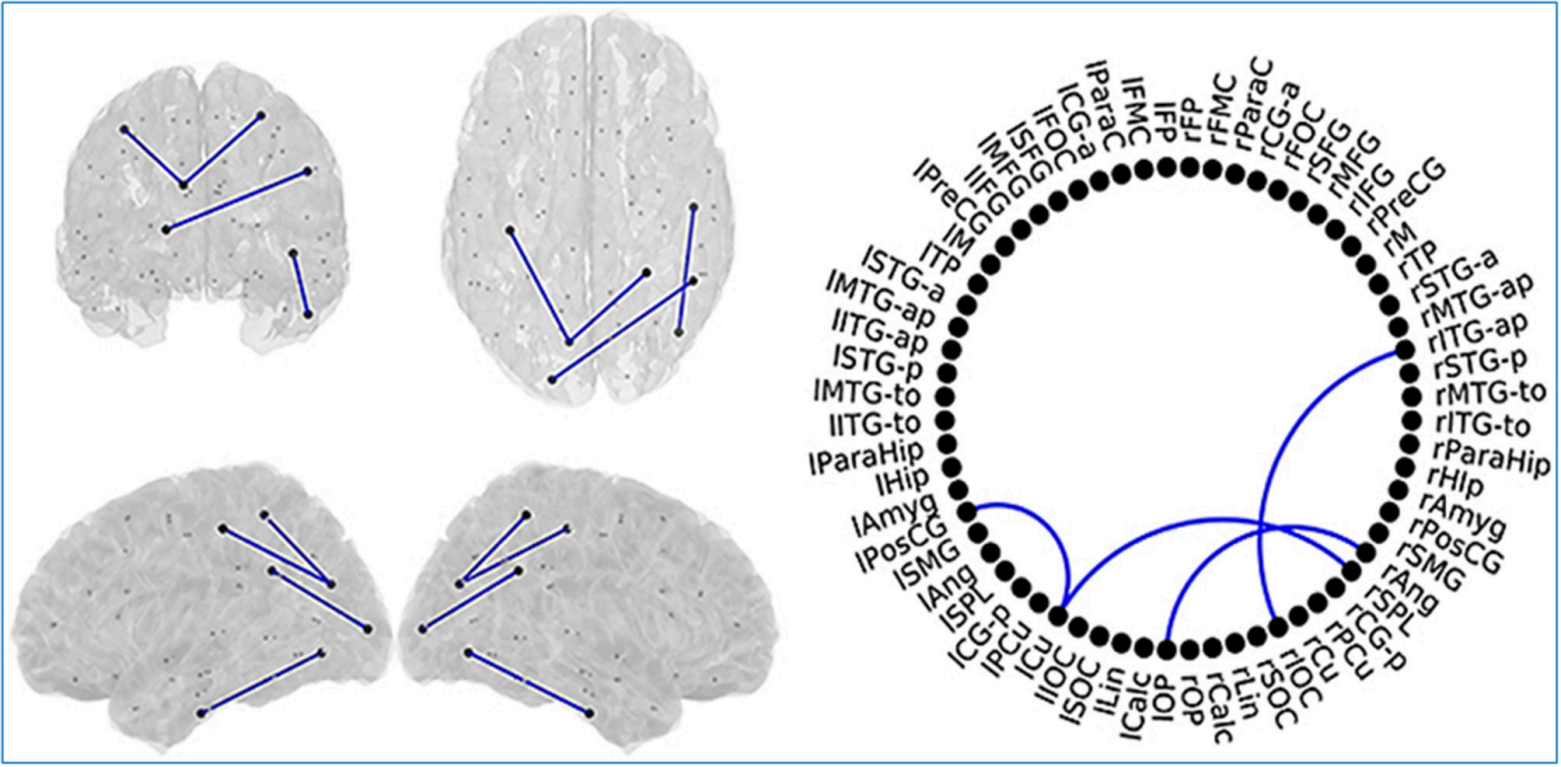
Links (connection among various brain regions) with significantly different FC values have been shown for comparative analysis. Left: Posterior, superior, left and right views of the brain. Right Circle plot shows a schematic view of the significant links. Comparison between subjective cognitive decline (SCD) and mild cognitive impairment (MCI) groups where Blue lines indicate a decreased FC-value in MCI respect to SCD. This Figure is taken from literature (López-Sanz et al., 2017) with permission.

MCI patients showed a network comprising four links where FC values were significantly lower compared to SCD FC values. This network with reduced FC connected temporal, parietal and occipital regions of the brain, and comprised both intra and inter-hemispheric links (Figure 8).

Both SCD and MCI groups exhibited a very similar spatial pattern of altered links: a hyper-synchronized anterior network and a posterior network characterized by a decrease in FC. This decrease was more pronounced in the MCI group. These types of FC alterations may work as a key feature to provide a useful tool to characterize the early stage and predict the course of AD.

In Table 1, we have listed some of the MEG studies, to gather information regarding the efficacy of MEG data analysis methodologies statistically. Accuracy, sensitivity, specificity and area under the region of convergence (ROC) curve (AURC) are briefed in this Table. Different studies have implemented different parameters for MEG data analysis. It is global analysis in most of the cases and not region specific. In addition, frequency bands are also varying in the experiments. AURC values reported in literature within 0.529 and 0.912 range for different studies. From overall observation we noticed that some common parameters like mean frequency (MF), sample entropy (SampEn), and Lempel–Ziv complexity (LZC) have exhibited accuracy ranging between approximately 77–85, 58–70, and 61–83%, respectively. Although high sensitivity and specificity have been accomplished in most of the studies, but it is difficult to draw any exact conclusion from these results due to the heterogeneity and diversity of the metrics. Hence, more region specific, frequency band specific studies are required to be executed to infer more useful information from MEG data analysis.

**Table 1:** Compilation of MEG data analysis using various methods (e.g., sample entropy, cross mutual information function etc.) along with specific frequency bands (δ, θ α, β, and γ), area of analysis (global and local), targeted population and corresponding statistical analysis values (e.g., accuracy, sensitivity etc.) is represented in this table.

**S. No**	**Type of analysis**	**Frequency band**	**Area of analysis (Global/Local)**		**Accuracy %**	**Sensitivity %**	**Specificity %**	**AURC**	**Population of Study**	**References**
1	Lempel-Ziv Complexity (LZC)	All	Global		83.33	80.95	85.71	0.9002	AD	Gómez et al., 2006a
2	Median Frequency (MF)	All	Global		85.37	85	85.17	0.912	AD	Poza et al., 2007b
	Individual Alpha Frequency (IAF)				80.49	80	80.95	0.821		
	Transition Frequency (TF)				73.17	70	76.19	0.760		
	Spectral Entropy (SE)				65.85	65	66.67	0.698		
	95% Spectral Edge Frequency (SEF95)				82.93	90	76.19	0.888		
3	Relative Power (RP)	β	Global		–	94%	67%	0.864	AD	de Haan et al., 2008
			R occipital		–	94%	78%	0.867		
4	Nonlinear Forecasting (NLF)	All	Global		76.7	80.0	73.3	–	AD	Gómez et al., 2008
5	Mean Frequency (MF)	All	Global		77.4	88.9	53.9	0.855	AD	Escudero et al., 2009
	Spectral Entropy (SpenEn)				61.3	55.6	69.2	0.727		
	Lempel-Ziv Complexity (LZC)				61.3	55.6	69.2	0.786		
	Spectral Entropy (SampEn)				58.1	72.2	38.5	0.645		
6	Spectral Entropy (SampEn)	All	Global		70.73	80	61.90	–	AD	Gómez et al., 2009a
	Lempel-Ziv Complexity (LZC)				78.05	80	76.19	–		
7	Detrended Fluctuation Analysis (DFA)	α1	Global		63.33	60.00	66.67	0.5511	AD	Gómez et al., 2009b
		α2			83.33	86.67	80	0.8400		
	Backward Detrended Moving Average (BDMA)	α1			80	80	80	0.8667		
		α2			73.33	60	86.67	0.6711		
8	Higuchi's Fractal Dimension (HFD)	All	Global		80	95.24	87.8	0.90	AD	Gómez et al., 2009c
			Local	Anterior	85	76.19	80.49	0.86		
				Central	70	90.48	80.49	0.87		
				Posterior	70	80.95	75.61	0.89		
				R Lateral	85	80.95	82.93	0.89		
				L Lateral	75	76.19	75.61	0.89		
9	Synchronization Likelihood (SL)	β1	Global		–	–	–	0.82	MCI	Bajo et al., 2010
		β2					0.72			
		γ					0.77			
10	Magnitude Squared Coherence (MSC) Cross Mutual Information Function (CMIFMA)	All	Local	Anterior	52.9 67.7	41.2 64.7	64.7 70.6	0.640 0.775	AD	Alonso et al., 2011
	MSC CMIFMA			L Lateral	55.9 76.5	58.8 82.4	52.9 70.6	0.512 0.813		
	MSC CMIFMA			Central	55.9 67.7	58.8 64.7	52.9 70.6	.536 0.771		
	MSC CMIFMA			R Lateral	52.9 76.5	58.9 76.5	47.1 76.5	0.529 0.903		
	MSC CMIFMA			Posterior	64.7 76.5	58.8 76.5	70.6 76.5	0.702 0.782		
11	Relative power	θ	Global		–	82	81	0.83	AD	Engels et al., 2016a

As observed from Figure 5, α band frequency in MCI changes, similarly in case of FC we observe the reduction in connectivity pattern for SCD and MCI from HC subjects. Also, from Table 1, it is clear that all these alterations in every parameter take place in AD as well as MCI. Henceforth, further research hit is required to unhide the concrete cause behind these variations. Though MEG studies found slowing down of brain rhythms in AD, but these findings cannot specify AD as majority of brain diseases show similar pattern of brain oscillations. Therefore, from single channel analysis studies it is inferred that a pattern is seen in AD irrespective of applied method. However, uniform distribution of slowing throughout the brain is not seen. These alterations in brain oscillations, the left parietal, occipital and temporal areas were found to be most frequently affected.

### Multimodal approach: MEG in combination with other imaging modalities

MEG findings reported a lower FC in AD than normal supporting the concept of AD as a ‘disconnection syndrome’ (Delbeuck et al., 2003; Koelewijn et al., 2017). These observations coincide with the results of PET, EEG, or fMRI studies (Leuchter et al., 1992; Besthorn et al., 1994; Desgranges et al., 1998; Adler et al., 2003; Greicius et al., 2007; Wang et al., 2007). Combining different modalities [e.g., EEG, PET, fMRI, magnetic resonance spectroscopy (MRS)] with MEG will be interesting (Langevin and Vachey, 1995; Horwitz and Poeppel, 2002; Rajapakse and Cichocki, 2002; Babiloni et al., 2004; Liu et al., 2006; Hall et al., 2014). It has been reported that integrated approach of EEG and MEG gives more accurate connectivity estimation than individually (Muthuraman et al., 2015).

It is observed that the integrated implementation of the high (millisecond) temporal resolution of MEG and EEG can give better accuracy than fMRI localization in measuring neuronal dynamics within well-defined brain regions and assessing the source localizing ability for identical stimuli (Sharon et al., 2007).

These methods are in fact independent of signal models and it makes them attractive for application in MEG analysis for rapid evaluation of data (Deo, 2015). Such approaches were utilized to joint multimodal processing of MEG and fMRI data. Processing of MEG data with the outputs of a deep neural network obtained from and trained on the same visual categorization task has been performed (Cichy et al., 2016a,b). With the help of these multimodal approaches, new principles of brain function, generalized to functional systems and patient population, are modeled (Cichy et al., 2016a,b).

There are certain neurochemicals (N-acetyl-aspartate (NAA) and myoinositol (mI)) alterations in AD. Concentration of NAA decreases and mI increases with the progression of AD. Integrated study of MEG with MRS has been performed to associate altered brain oscillations with neurochemical changes (Maestu et al., 2005). For a working memory task, the AD group showed a reduced number of activity sources over left temporoparietal areas in MEG analysis. In another MRS study increase in creatine, mI, and in the mI/NAA ratio was observed in bilateral temporoparietal region. These results were correlated with MMSE score and 65% of the variance was found (Maestu et al., 2005).

Modulation of gamma oscillations is a widely established mechanism in a variety of neurobiological processes, but its neurochemical basis is not fully known yet. In addition, research reports suggest that γ oscillation properties depend on GABAergic (gamma-Aminobutyric acid) inhibition (Kujala et al., 2015). The link between GABA concentration and gamma oscillations is a thrust area of research. A direct relationship between the density of GABA receptors and γ oscillations in human primary visual cortex (V1) has been established by an investigation by combining Flumazenil-PET (to measure resting-levels of GABA receptor density) and MEG (to measure visually induced gamma oscillations; Kujala et al., 2015).

Involving various neurotransmitters with altering brain oscillations will open a new research domain for clinical application and it is an important thrust area in our laboratory.

## Conclusions

MEG is a powerful technique for the recording of changing activities of brain functions. From single channel analysis, a patterned and consistent slowing of brain oscillations has been observed in AD. FC studies revealed decreased connectivity in AD than controls. EC studies must be implemented further to conclude anything. As a whole, association of parietal and temporal areas has been reported with the advancement of AD in comparison to HC subjects. Involvement of the hippocampus has also been demonstrated but more investigation is required.

The MEG is useful to bridge other electrophysiological measures like EEG, local field potential (LFP), fMRI, PET, and brain stimulation. It opens opportunities for the cross validation of the research findings from various modalities. Moreover, MEG in combination with appropriate modalities can be helpful to understand the nature of changes of the neurotransmitters like gamma-Aminobutyric acid (GABA), glutamate etc. (Muthukumaraswamy, 2014; Kujala et al., 2015). MEG has the capability to open up new avenue for clinical research in AD.

MEG data is usually recorded in altered investigational environments, having a spatiotemporal configuration, sampled at several sensors and multiple time points. Researchers aim to recognize the alteration among the data observed in these conditions. The processing and analysis procedure varies in research studies as can be observed from Figures 3, 4. Some studies work on sensor space data and some on source space which introduce the major alteration in processing pipeline and also the frequency bands considered for the study differs as per requirements and anticipated outcomes. Moreover, in most studies the conditions vary with respect to the configuration of stimulus being presented instantaneously before or during the registration of the signal. In other studies, the conditions change with respect to the type of response given by the experiment subjects. Another aspect in this regard is that AD population is diverse with respect to gender, age, demography, disease progression etc. are difficult to be found similar among studies. Modality Integration also plays an important role in experimental design for AD research. Various modalities such as EEG, fMRI, PET, DTI have been integrated with MEG to gain complementary information. Data availability for AD research is also another major research avenue to investigate for AD research.

Hence, majority of the MEG based brain connectivity studies using same experimental pipeline in AD research cannot directly be compared. It necessitates the demand for open science in MEG research by generalizing a specific pipeline for MEG data acquisition, processing and analysis in clinical setting. Research groups should be interested in making research accessible for all to share their materials and data, others can use and analyze them in new ways, potentially leading to new discoveries. This will reduce the so-called “reproducibility crisis.” MEG data being available will be scientifically beneficial for researchers that can open new avenue in this realm of AD research.

## Author contributions

PKM conceptualize the idea, literature search, and manuscript writing. AB literature search, writing, and figure preparation. MT literature search and writing manuscript. AS literature search and writing.

### Conflict of interest statement

The authors declare that the research was conducted in the absence of any commercial or financial relationships that could be construed as a potential conflict of interest.
